# Red emitting carbon dots: surface modifications and bioapplications

**DOI:** 10.1039/d3na00469d

**Published:** 2023-08-03

**Authors:** Dawson Benner, Pankaj Yadav, Dhiraj Bhatia

**Affiliations:** a Department of Engineering, Texas A&M University College Station 77843 Texas USA; b Biological Engineering Discipline, Indian Institute of Technology Gandhinagar Palaj 382355 Gujarat India dhiraj.bhatia@iitgn.ac.in

## Abstract

Quantum dots (QDs), and carbon quantum dots (CDs) in particular, have received significant attention for their special characteristics. These particles, on the scale of several nanometers, are often produced using simple and green methods, with naturally occurring organic precursors. In addition to facile production methods, CDs present advantageous applications in the field of medicine, primarily for bioimaging, antibacterial and therapeutics. Also, CDs present great potential for surface modification through methods like doping or material mixing during synthesis. However, the bulk of current literature focuses on CDs emitting in the blue wavelengths which are not very suitable for biological applications. Red emitting CDs are therefore of additional interest due to their brightness, photostability, novelty and deeper tissue penetration. In this review article, red CDs, their methods of production, and their biological applications for translational research are explored in depth, with emphasis on the effects of surface modifications and doping.

## Abstract 2

Steps involved in red CD use begin with the synthesis methods chosen. Surface modification is carried out either during synthesis or afterwards; following this process, the CDs can be used for many biological applications.

## Graphical abstract

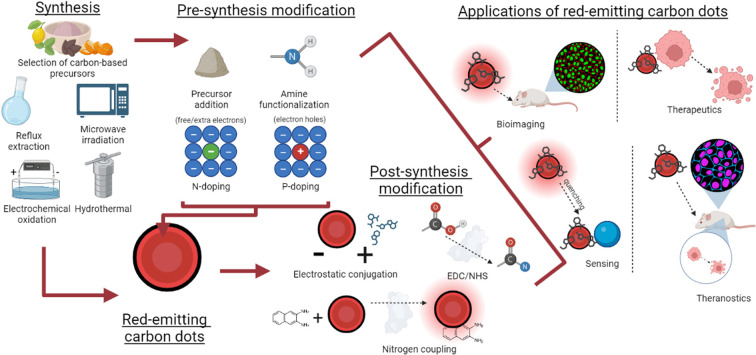

## Introduction

QDs are quasi-spherical zero-dimensional nanomaterials, meaning they are below 100 nanometers in all three dimensions. When illuminated by UV light, they excite an electron to a higher energy state, resulting in the emission of energy as a wavelength of light.^1,2^ Due to this unique behavior and small size, QDs were of significant interest in the field of semiconductors.^3^ However, their applications were soon expanded to medical use as an imaging agent for a variety of organs in animals.^4^ Naturally, this necessitated greater focus on biocompatibility and reduced cytotoxicity, ruling out traditionally used elements like cadmium, which can cause cell death.^5^ However, a chance discovery by Xu *et al.* when purifying nanotubes resulted in CDs coming to the forefront of the medical field.^6^

Carbon has obvious benefits of improved biocompatibility, and its prevalence in the natural world opened many new avenues for synthesis when compared to heavy metal-based dots. For example, lemon peel waste has been used by Tyagi *et al.* to synthesize CDs for use in photocatalysis and sensing.^7^ Just as with synthetic material based QDs, CDs have been shown to produce a variety of different wavelengths when excited. Bao *et al.* have shown a method to create CDs in a range of colors from the same precursor (*Eleocharis dulcis*) by changing the hydrothermal temperatures at which the production took place. These dots served obvious purpose as tunable fluorescent agents and possess an ability to detect Fe^3+^ ions as well.^8^ In addition, blue CDs have been successfully prepared from low-grade coal with an ability to sense silver ions and obvious use in imaging.^9^ However, most dots emit in the blue range and possess lower efficiency than traditional QDs. Also, blue/green CDs have been shown to cause autofluorescence in cells.^10^ These two factors primarily necessitate the synthesis of red, or near-infrared, CDs, a topic of high demand.

In addition to the increased focus on red CDs, there has been significant interest in doping quantum dots. This is a procedure in which impurities are intentionally introduced during the synthesis process to enhance various qualities of CDs. Nitrogen is typically used as a dopant in this process due to its widespread availability and improved effect on photoluminescence.^11^ In short, N-doping, coincidentally carried out by nitrogen introduction, adds an extra electron to the QD which acts as an easily excitable negative charge carrier, hence the N. P-Doping, on the other hand, adds one too few electrons, creating a functionally positive hole which moves opposite to electrons and is the reasoning for this method's name.^12^ Both strategies increase conductivity and are expanded upon in greater detail in a later section.

Similarly, to doping, surface modification is a technique which has great potential to change and enhance the behavior of QDs in a variety of ways. The most common method is material mixing, in which precursors are chosen in a manner that leads to modification during CD production. For example, Yin *et al.* used hyperbranched polymers to functionalize CDs that can respond to multiple stimuli, proving advantageous in both imaging and delivery of organic molecules.^13^ The introduction of other materials than carbon brings about new behaviors, presenting a wide range of potential to be explored.

To effectively evaluate the state of research on red-emitting CDs and surface modifications, it will be important to consider the processes by which CDs are produced and the applications for which CDs are used. The steps of synthesis for CDs, and varying methods, will be explored, as well as the techniques for analysis, characterization, and testing of CD behaviors. The processes of N/P-doping and surface modification of CDs are then explored, looking into the applications of pre- and post-synthesis modification. Finally, current findings on longer-wavelength CD applications in bioimaging and treatment of diseases are examined.

### Synthesis of red CDs

Red CD production follows either top-down or bottom-up synthesis. Top-down operations typically involve carbon materials being broken down by primarily chemical and electrochemical processes. Dots have been formed from purification following the application of thermal energy to single-walled carbon nanotube fragments by electrophoresis; these particles varied in size and had yellow-orange emission properties.^6^ Top-down methods also often use filtration and centrifugation to purify their final product.^14^ Additionally, an electrochemical procedure involving cycling voltage has been described to synthesize blue-emitting CDs.^15^ While both methods successfully produce fluorescent CDs, there are obvious drawbacks to the top-down approaches of synthesis. Quantum yield (QY) is an important measurement and informally described as the number of photons emitted compared to the number of photons absorbed. Low QY was reported in both papers, with Xu achieving 1.6% and Zhou's procedures producing QY of 6.4%. In addition to low QY, only around 10% of material produced in these methods had QD characteristics, indicating an inefficient method of production.^6^ Thus, bottom-up synthesis processes have been of greater interest.

Bottom-up methods of synthesis come in several forms, each offering advantages over top-down synthesis. Among these, microwave irradiation is particularly interesting due to its low-cost, rapid nature.^16^ This process involves precursors added in solution (typically water or ethanol) which is then heated in a microwave; additional steps usually involve purification by dialysis or centrifugation.^17^ This process is also of interest due to the wide range of precursors which can be used, especially organic/waste molecules like coffee grounds.^18^

Attention must be paid to the precursors used during CD synthesis, especially when surface modifications are sought after. One common method is the use of two different precursors, one which supplies carbon and one which supplies another atom. A reaction between acetonitrile and sodium-naphthalene was carried out and shown to produce red CDs. Thanks to their nitrogen-doped quality, the dots possess narrow-band excitation-independent emission at 588 nm; they can easily be modified to be hydrophilic and thus optimal bioimaging agents.^19^ Similarly, Karakoçak *et al.* crafted N-doped CDs using citric acid and aliphatic diamines in various ratios. In addition to the benefits presented above, these CDs possessed tunable emissions and a high degree of control over size.^11^

Other elements have been successfully introduced through precursor choice. Ge *et al.* successfully created sulfur-doped CDs using similar methods with polythiophene phenylpropionic acid as a precursor.^20^ This CD has advantageous properties in photothermal therapy and tumor imaging, showing the importance of surface modifications. In fact, CDs have been successfully enhanced in this way with multiple atoms, as shown by Lan *et al.* Through a hydrothermal treatment of diphenyl diselenide and polythiophene, they co-modified CDs with selenium and sulfur. These CDs, while platelet shaped, targeted HeLa cells with photothermal therapy.^21^

Additionally, the choice of solvent has a proven effect on the characteristics of resultant CDs. Using differing ratios of dimethylformamide, ethanol, and water, CDs emitting blue, green, yellow, and red wavelengths were synthesized.^22^ The latter was created using pure dimethylformamide as a solvent and 1,3,6-trinitropyrene as a carbon precursor and reported high yields of 92%; this occurs because dimethylformamide inhibits the formation of insoluble byproducts. By using normalized polarity parameters (based on interactions between solvent and target)^23^ for ten different solvents,^24^ it was discovered that increased relative solvent polarity generated binaphthyl-based CDs with higher absorption and emission wavelengths. This occurs because the nitrogen modified on the surface of these CDs easily attract charges present in the surrounding higher polarity solvent.^25^ Dipole moments are also stronger in polar solvents, which means they require more energy to switch direction. As a result, the charge carriers are more likely to be attracted to the nitrogen present on CDs rather than to a solvent molecule.

**Fig. 1:**
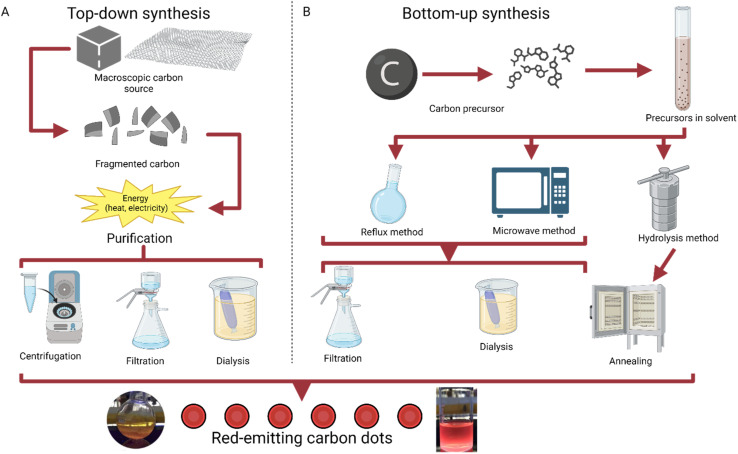
Graphical explanation of top-down and bottom-up synthesis.^29^ (A) Methods of top-down synthesis, going from a macroscopic carbon source to carbon dots.^30^ (B) Methods of bottom-up synthesis, working from a molecular carbon precursor to carbon dots through reflux, microwave synthesis, or hydrolysis.^31,32^

The method of bottom-up synthesis has a general set of steps that are usually followed. A carbon precursor is first selected, usually from an organic source like lemon peel or coffee grounds.^26,27^ Then, the precursor is put into a choice of solvent, typically after drying or powderizing. Next, a method of temperature increase is selected, typically reflux, microwave, or hydrolysis.^28^ Hydrolysis methods are followed with purification *via* annealing, while reflux and microwave synthesis can use multiple filtration techniques, including filtration and dialysis; this step yields the product of carbon dots (Fig. 1).

### Surface modification

#### Pre-synthesis approaches

##### N-Doping

**Fig. 2:**
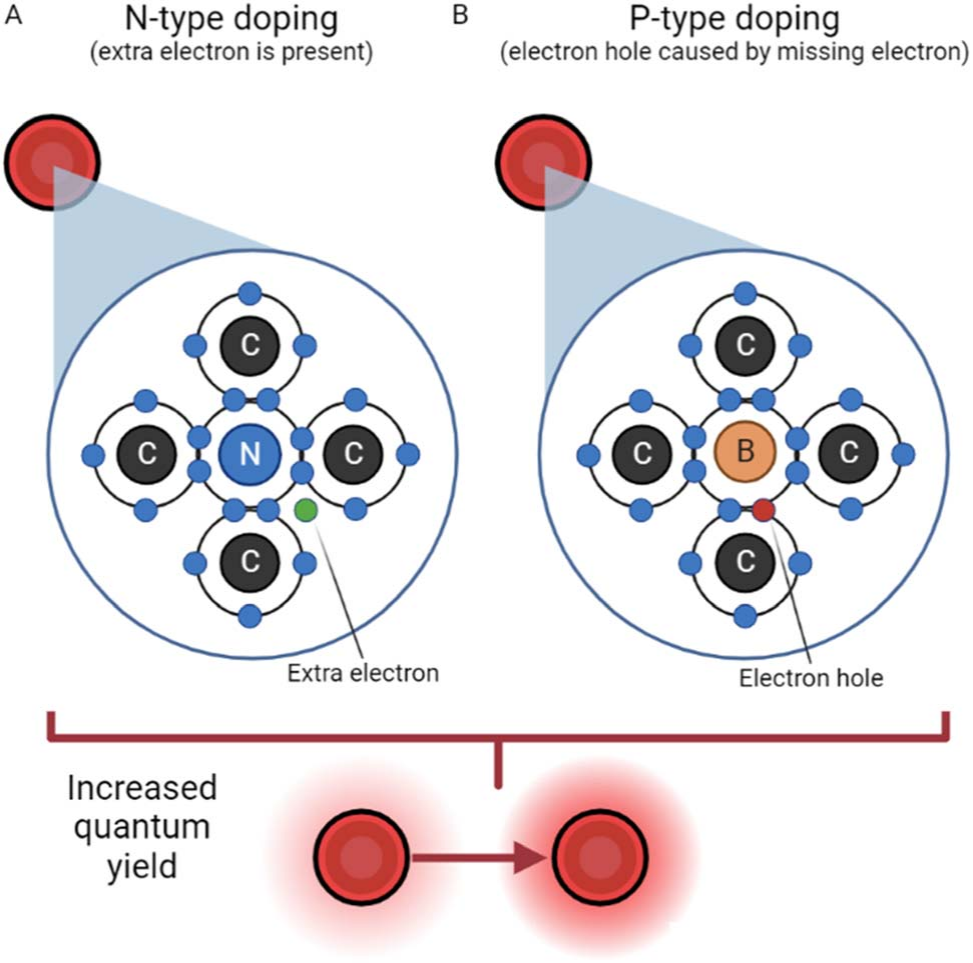
N-Type doping *vs.* P-type doping.^33^ (A) N-Type doping, reliant on the presence of an extra electron in the carbon dot structure. (B) P-Type doping, where an electron hole acts as a positive particle.

Doping is a strategy often used for semiconductors and traditional QDs due to the ability to greatly improve conductivity; naturally, it has applications to the field of CDs as well. Doping is described as the intentional introduction of impurities to a semiconductor or dot with the intent of improving qualities like conductivity, fluorescence, and quantum yield. The process of N-doping is often carried out using nitrogen (because of its five valence electrons). N-Doping covalently bonds four of nitrogen's valence electrons with the four valence electrons from carbon, leaving one free charge carrying electron from nitrogen which can be seen in Fig. 2. This requires less energy to be excited and greatly increases the luminescence properties of CDs.^33^ The name N-doping comes from the fact that the free electron provides a negative surface charge.

As mentioned previously, N-doping has been used to create CDs with sensing capabilities, specifically for Fe^3+^ ions.^8^ These dots possess tunable emission wavelengths, and the N-doping lends them a lower detecting sensitivity, proving useful in detection of potentially harmful ions. Additionally, Yang *et al.* used this process to generate excitation-independent red emission agents, critical for bioimaging.^19^ The N-doping contributes to their intensity, which will contrast well with natural fluorescence. N-Doped CDs have also recently been produced to accomplish both goals at once, detecting IO_4_^−^ ions well below standard limits and providing *in vivo* imaging.^34^ This example highlights the need for effective doping strategies, as by simply introducing other elements during fabrication, the traits of CDs can be enhanced greatly. Facile methods have been detailed to produce N-doped CDs from natural materials (citric acid) and ethylenediamine, indicating that the technique is both cost-effective and useful in practice.^11^ Low cytotoxicity remains a constant among N-doped CDs, proving they are more viable for medical use than their metal-derived precursors.

##### P-Doping

In a somewhat reversed process, P-doping requires an atom with three valence electrons, like boron. In this process, however, boron has one too few valence electrons to bond covalently with carbon. As a result, an electron hole is created with a functionally positive charge, depicted in Fig. 2. This hole's behavior has been described, in a simplified manner, as an air bubble in a water bottle, moving the opposite direction as electrons.^35^ The hole attracts electrons from another atom, leaving a hole in another atom, and the process repeats. Thus, P-doping increases conductivity and luminescence by creating a positively charged quasiparticle.

Boron has, interestingly, been used as a co-dopant with nitrogen to enhance the CDs reported on previously.^34^ This was done to further increase the sensitivity and detection range, yielding a linear response to IO_4_^−^ in the form of fluorescence quenching. Further exploration of co-doping with nitrogen and boron produced red-emitting CDs which are capable of targeting lysosomes, an organelle crucial to cell survival and internal balances (Deng *et al.*, 2023).^36^ These CDs boast improved QY and highly specific targeting abilities which can be used translationally for apoptosis. Additionally, the tracking ability has been traced back to the addition of boron as a dopant, indicating the powerful role played by doping CDs. High QY boron-doped CDs have been tweaked to yield either highly fluorescent red or green emissions, dependent on the concentration of boron in the production process.^37^ This highly scalable framework and P doping both have major implications for the field of biosensing.

**Fig. 3:**
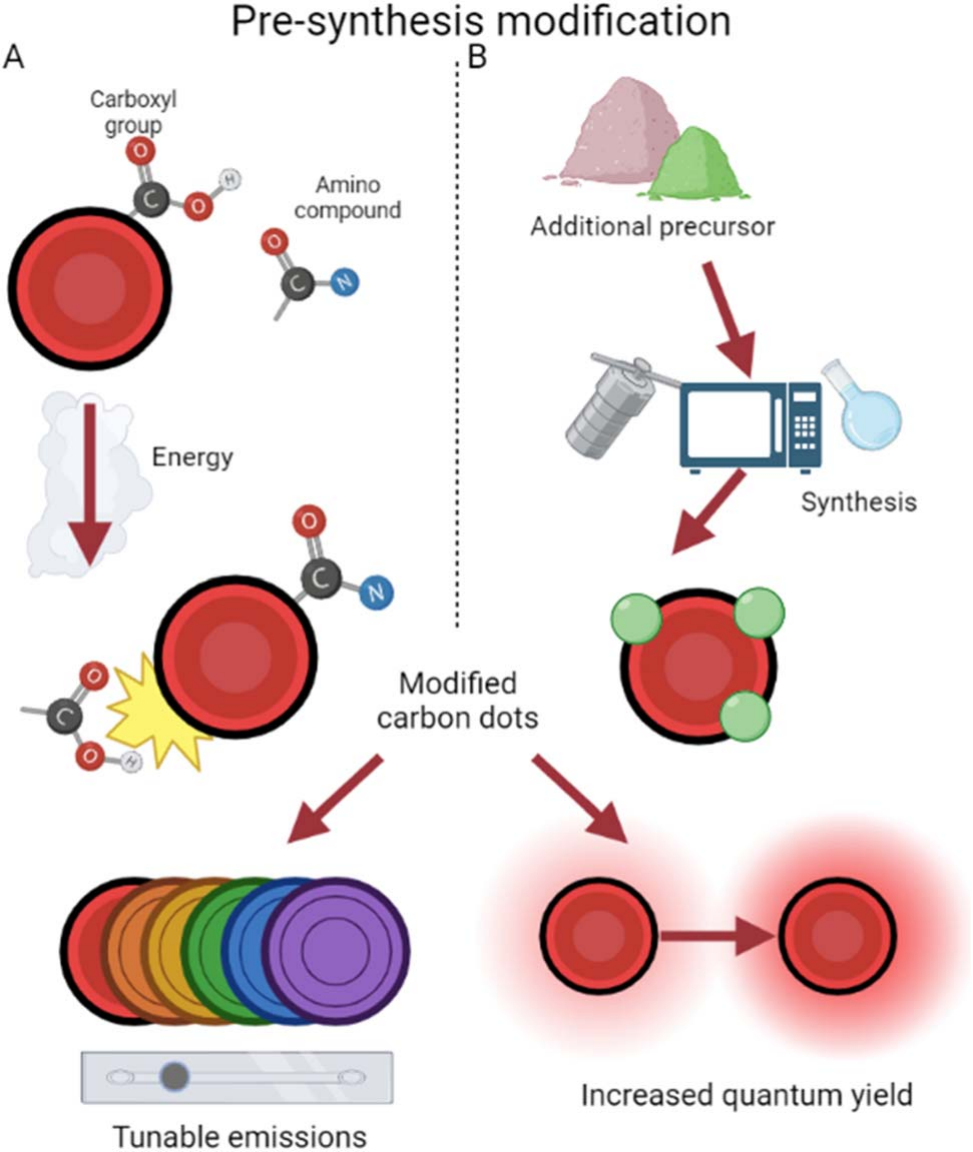
Pre-synthesis surface modification approaches. (A) The affinity of CDs for amino compounds during synthesis results in modified CDs.^38^ (B) The choice of an additional precursor often modifies CDs during production.^46^

Since CDs contain abundant amino/carboxyl groups on their surfaces, they can amidate easily with amino compounds as ligands for surface modification,^38^ a process depicted in Fig. 3. This has been used to increase quantum yield and fluorescence emission by preventing the recombination of electron–hole pairs.^39,40^ QY has increased from 1.3% to 3% using these methods, and amino group modification ensures biological compatibility. Additionally with an imaging focus, surface modification has been demonstrated to enable tunable wavelengths in CDs. Glucose-synthesized CDs were prepared in the presence of monopotassium phosphate, resulting in particles which emit different wavelengths depending on the phosphate concentration.^41^ By increasing the phosphate concentration, particle size increases, along with wavelength emitted as a result. Amine group functionalization has also been further utilized by Hao *et al.*^42^ to boost quantum yield and fluorescence. This study prepared CDs from tris(hydroxymethyl) aminomethane, subsequently modifying the surface with betaine hydrochloride and distributing them in a polyethylene glycol (PEG) film. This process creates CDs which are dispersible in water,^43^ while the PEG film stabilizes surface sites and reduces radiative recombination,^44^ thereby increasing quantum yield. In addition, by tweaking the weights of PEG added, Hao *et al.*^42^ tuned emissions and synthesized high yield red CDs.

In addition, CDs have been surface modified by the introduction of other non-amine ligands during production. This occurs with the selection of precursors containing the desired modifier elements (Fig. 3). For example, CDs have been prepared from *Hypericum perforatum* and tuned *via* the changing of reactive solvents, resulting in regulated quantum yield and different reactive oxygen species (ROS) generation.^45^ This helps to overcome current chemotherapy limits of one-typed photothermal therapy and improves efficiency even under low-oxygen tumor environments. By use of selenocystine as a precursor, Zhou *et al.* were able to synthesize selenium-functionalized CDs which, due to functionalized α-carboxyl groups, function like large amino acids and inhibit amyloid-beta aggregation.^46^ Amyloid-beta assemblies are a hallmark of inhibited neurological activity,^47^ and by surface modification, these CDs act like leading Alzheimer's disease treatment.^48^ The enlarged size of these conjugates also results in their emission shifting closer towards red, lending them potential in simultaneous imaging and therapy for Alzheimer's disease. CDs have been produced and modified on the surface with hexadecylamine ligands, revealing that smaller ligands resulted in easier electron transport and thus higher conductivity.^49^

#### Post-synthesis surface modification approaches

**Fig. 4:**
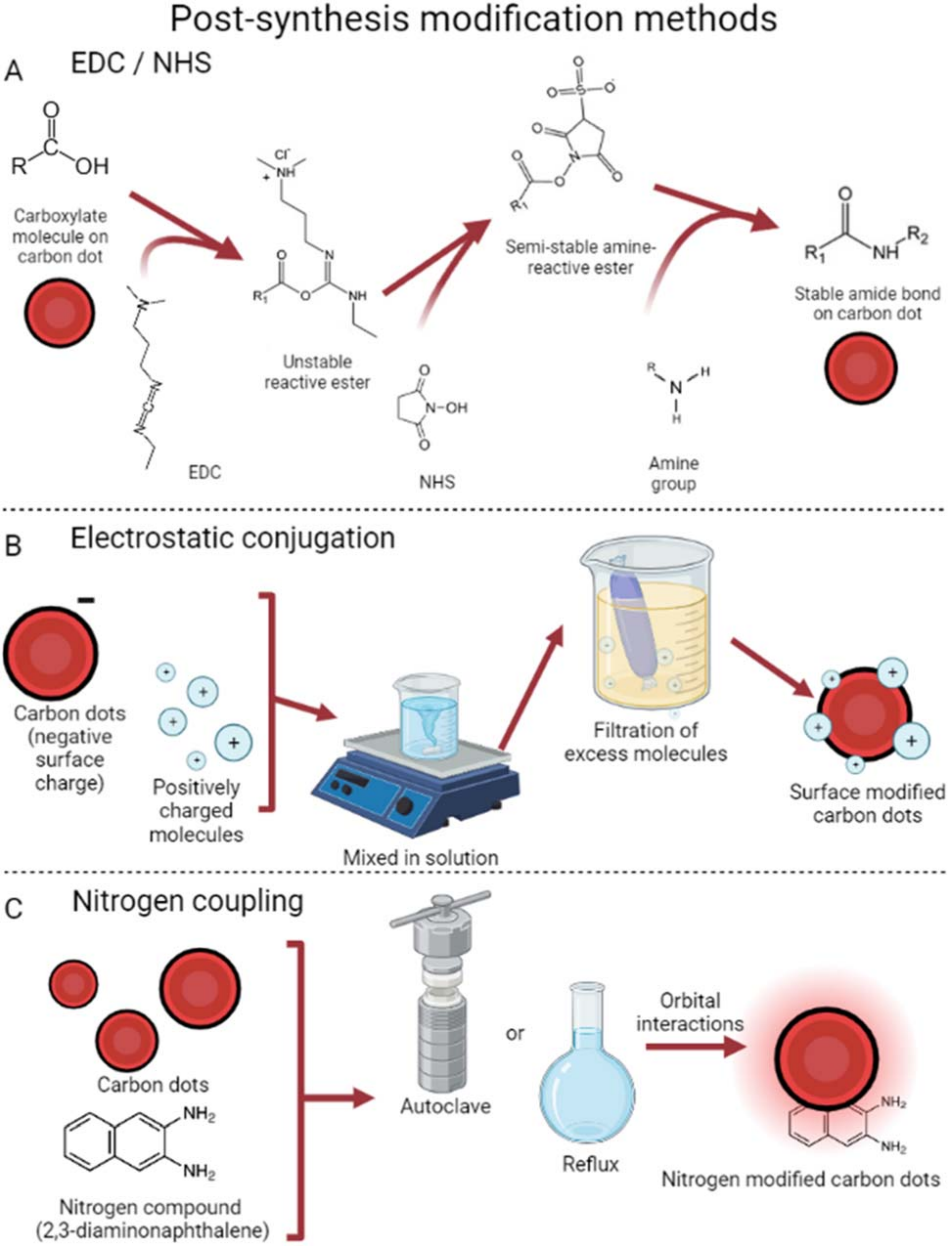
Post-synthesis surface modification. (A) EDC/NHS reactions are used to activate CDs for subsequent modification with larger amine groups.^70^ (B) Electrostatic conjugation uses surface charge differences for bonding.^59^ (C) Nitrogen coupling relies on orbital interactions to attach nitrogen compounds to CDs.^65^

Post-synthesis surface modification is often used to introduce more complicated molecules to CDs once their size and other traits have been properly tuned.^50^ One leading technique is a reaction which is abbreviated as EDC/NHS and shown in Fig. 4, resulting in covalent surface coupling between a carbon dot and an amino group.^51^ Named after the chemicals ethyl(dimethylaminopropyl)carbodiimide (EDC) and *N*-hydroxysuccinimide (NHS), the process requires a precursor with a carboxyl group (CDs) and an amino group which is later reacted.^52^ The carboxyl group reacts with EDC to form an unstable reactive ester; next, NHS bonds, forming an amine-reactive ester. Afterwards, the free amino group attaches to form a stable amide bond; this process is often used to attach compounds with amino groups to the surface of CDs.^50^ EDC/NHS has been used by Zheng *et al.* to attach oxaliplatin, a chemotherapeutic, to the surface of highly luminescent carbon dots, creating effective theranostic agents.^53^ Quinoline derivatives have been conjugated with CDs for sensing of zinc,^54^ showing potential for EDC/NHS as a tool to enhance biosensing agents. Similarly, EDC/NHS successfully conjugated CDs with oligodeoxyribonucleotides as fluorescent mercury ion monitors; the quenching of CD fluorescence allowed for highly selective and low-limit sensing.^55^ In another direction, Disha *et al.* used EDC/NHS to covalently attach the progesterone monoclonal antibody to a CD, with the intent of detecting progesterone levels.^56^ They used fluorescence resonance energy transfer with graphene oxide as a quencher; when in the presence of progesterone, the graphene is displaced, and fluorescence turns on.^57^ While studies in this area are limited, these results show potential for antibody-functionalized CDs for detection of hormones or antigens on cancer cells, viruses, or bacteria.

Another common method of post-synthesis modification is based on surface electrostatic interactions^58^ which rely on differences in surface charges to conjugate CDs and the desired surface modifier. This method, visualized in Fig. 4, tends to require fewer steps to use and is thus very popular. With the use of electrostatic interactions, doxorubicin has been successfully attached to CDs.^59^ Due to the nitrogen groups on CDs synthesized from 4-aminophenol and potassium periodate,^60^ targeted drug delivery was achieved successfully. This technique takes advantage of small CD size to reduce cancer cell viability to 21% (*vs.* 50% with free doxorubicin) by penetrating the cell nucleus.^61^ Additionally, these dots provide their traditional deep red fluorescence, enabling strong visualization within the tumor 12 hours after injection. Conjugation has also been achieved with cisplatin, a normally neutrally charged molecule, by attaching a Pt(iv) prodrug instead.^62^ This molecule becomes cisplatin in the reducing environment of cancer cells,^63^ both reducing effects in benign cells^64^ and enabling covalent CD conjugation.

Nitrogen coupling has also been used in a variety of ways to modify the surface of CDs.^65^ This strategy allows for modification of the highest occupied/lowest unoccupied molecular orbitals, which in turn causes tunable emission wavelengths and quantum yield. Carbon dots were synthesized which, once coupled with diaminonaphthalene, exhibited increased red emissions.^66^ This process took synthesized graphene carbon dots and placed them with diaminonaphthalene in an autoclave at 180° for 12 hours. Dubbed DAN-GQDs, the resulting dots also exhibited the highest quantum yield in their study, at 15.4%.^65^ These nitrogen-coupled particles still possess low cytotoxicity, showing potential for nitrogen to enhance imaging applications.^67^ Numerous other methods exist involving nitrogen coupling, all achieving similar results of enhanced photoluminescence and boosted quantum yield. Interestingly, Babar *et al.* were able to create explosive-detecting by the addition of nitrogen post-CD production.^68^ This method refluxed a mixture of CDs (synthesized from dextrose^69^) and HNO_3_, later decanting off the nitric acid and leaving behind nitrogen-coupled CDs. These dots showed linearly quenched fluorescence in the presence of increasing concentrations of trinitrophenol, a dangerous explosive, further showing the widespread applications of post-synthesis CD surface modification.

### Applications of red CDs

#### Bioimaging

The current state of red CDs in bioimaging relies on their red/NIR properties when excited and the fact that these molecules tend to avoid autofluorescence in cells.^10^ Confocal microscopy is one of the most successful methods at capturing high-quality imaging in thick tissue or of small particles.^71^ Laser light is directed at differing frequencies using a microscope so that each color (red, green, and blue) can be isolated. This ensures the red CDs can be viewed clearly in contrast to other cell parts which may be treated with dyes.^72^ One trait sometimes sought after that red CDs offer is wavelength-independent emission;^73^ however, many CDs can be ‘turned’ red because they possess tunable emissions. While exhibiting wavelength-dependent emission, CDs synthesized by Hua *et al.* have shown to accumulate in the mitochondria, giving them potential for mitochondrial imaging.^74^ Synthesized from chitosan, ethylenediamine and mercaptosuccinic acid in varying ratios, these dots almost exclusively stained mitochondria due to their positive charge and lipophilicity. Additionally, Zhang *et al.* synthesized CDs with emission wavelengths of 680 nm which have a propensity to accumulate in tumors.^45^ Excitation dependent, deep red-emitting CDs have been synthesized from citric acid and ethylenediamine and were able to diffuse to the cornea, providing potential for ocular bioimaging.^11^

The presence of surface modifiers often takes red CD fluorescence and applications to a new level. The addition of fluorine increased the pH range of stability, red shifted emissions, and improved fluorescence properties.^75^ Fluorinated polymers are more likely to associate with themselves because fluorine decreases the surface energy of most polymers;^76^ this synergistic effect improves their visibility in bioimaging. Amine surface modifications have been used to increase the specificity of CDs, endowing them with increased targeting abilities. For example, high QY (84%) CDs have been functionalized with the use of an amine precursor for imaging specifically in the lysosome.^77^ These CDs also emit a narrow range of wavelengths when excited with varying emissions, making them easily distinguishable in imaging applications. This behavior has been replicated with additional boron and nitrogen doping,^36^ further enhancing the visibility of CDs. The pathway of clathrin-mediated endocytosis^78^ is believed to influence uptake of CDs *in vivo*, explaining their increased endocytosis in kidney and liver. This pathway, in conjunction with amine modified red CDs, has enabled ultra-fast (40 seconds) imaging of lysosomes.^79^ Monitoring lysosomes is important for understanding the breakdown of macromolecules,^80^ neurodegenerative diseases,^81^ and autoimmune diseases (due to their influence on the production of cytokines).^82^ Thus, surface modifications of CDs with amines may prove beneficial to multiple avenues of medical research.

#### Therapeutic applications

A major area for focus with red CDs, due to their ability to penetrate tissue well, is the treatment of diseases, especially cancer. Photothermal therapy (PTT) is a field which involves artificially increasing the cell temperature to trigger cell death, or apoptosis.^83^ Typically, a photothermal agent is administered and ‘activated’ in the presence of NIR light. However, traditional PTT agents encounter issues when they cause death to non-cancerous tissue. CDs take advantage of enhanced permeability and retention (EPR) of tumors^84^ and use their small size to accumulate passively in tumors. Bao's nanoparticles, synthesized with bottom-up methods from citric acid, DMSO, and urea, emit at 720 nm and are successful PTT agents due to their heat generation when excited (X. Bao *et al.*, 2018).^85^ Lan *et al.* have also crafted NIR CDs, though from diphenyl diselenide and polythiophene, with similar PTT success.^21^ These CDs, which end up co-doped with sulfur and selenium, targeted HeLa cells and inhibited tumor growth. More recently, photodynamic theory (PDT) has been administered using red CDs to produce reactive oxygen species (ROS).^45^ In this method, CDs are activated by the appropriate wavelengths of light, exciting oxygen species into ROS which bring about apoptosis.^86^ However, tumors often have low oxygen environments,^87^ something overcome by Zhang's CDs. Even in tumor hypoxia environments, these CDs generated ROS sufficient to inhibit tumors without damage to other organs in the mice.^45^ This presents a significant advance and application for chemotherapy with red CDs.

Red CDs have also been synthesized with the purpose of delivering drugs. For example, Hu *et al.* were able to use Osmanthus seeds to synthesize red CDs which were later bonded to cisplatin *via* surface modification.^62^ This anticancer drug was then released in slightly acidic, high glutathione environments, both of which are hallmark tumor traits. Naturally, targeted delivery reduces the common side effects of extraneous tissue death when administering chemotherapy. Similarly, work has been done using doxorubicin loaded onto the surface of CDs for targeting cancer cells and cancer stem cells.^88^ CDs were synthesized by heating 4-aminophenol and potassium periodate, and doxorubicin was attached due to favorable charge interactions. The drug-modified CDs were then able to penetrate the nucleus of malignant cells, reducing tumors to 1/6 of their initial size. Red CDs have been used to encapsulate liposomes and trace the anticancer agent cinobufagin, additionally increasing the efficiency of cinobufagin.^89^ This increased photoluminescence by up to five times, and it takes advantage of the well-researched behavior of liposomes as drug delivery devices.^90^ However, it is by modification with red CDs that this approach achieves more efficient results. This effect has been replicated in additional studies, utilizing the fluorescence of CDs as a method for tracking doxorubicin *in vivo*.^91^ This takes advantage of the covalent bonding between doxorubicin and CDs, enabling localized treatment of cancer.

CDs with near-infrared irradiation have also been developed for boosted therapeutic efficiency.^92^ In this experiment, the long wavelength allows for fluorescent and photoacoustic imaging while also triggering tumor ablation with sufficient photothermal energy. These hold the additional benefit of many chemotherapeutic red CDs of reduced non-tumor cell death, indicating a promising agent for translational research. In fact, significant research has been conducted with the goal of further enhancing near-infrared CDs as chemotherapeutic agents. These particles, often doped by other atoms on their surfaces,^93^ take advantage of enhanced photothermal conversion to deliver targeted tumor death. These approaches are facilitated by the small particle size, which allows increased tumor uptake^94^ and therefore reduced side effects. In addition, a variety of carbon sources have been used successfully, including polymers, NIR dyes, and porphyrin derivatives,^95–97^ demonstrating that chemotherapeutic properties are not unique to one specific precursor and its use in synthesis. The wide range of approaches to red CD use in chemotherapy is promising for researchers, but it also indicates that there is significant room for improvement in terms of optimizing and honing results.^98^

#### Sensing

Another common application of CDs is the detection of various harmful elements in trace amounts, usually by the mechanism of fluorescent quenching. This process decreases CD visibility by either dynamic or static quenching.^99^ Dynamic quenching involves transfer of an electron from a donor (in this case CDs) to an acceptor, which is the ion being detected. Static quenching, however, happens when a compound forms between the fluorophore and its quencher. These two methods together explain interactions between ions and CDs that enable their detection, an enticing quality for particles which are harmful even in trace amounts.

For example, chromium(iii) sensing has been facilitated by red CDs which were synthesized from wine lees, a residual from wine production.^100^ These dots function by emitting fluorescence, at around 675 nanometers, until the presence of a chromium(iii) ion quenches this fluorescence. The dot also exhibits a linear relationship between pH and intensity. Additionally, through the power of surface modifications, Wu *et al.* created boron/nitrogen co-doped dots that respond to periodate (IO_4_^−^).^34^ This method boasts a lower detection limit than current standards and a remarkably high QY of ≈87% while following the green methods described earlier. While yellow green in color, CDs have been synthesized to detect phoxim, a harmful pesticide, below the regulation levels; this method is also more cost efficient and faster.^101^ Interestingly, red CDs have been synthesized with multi-ion detection properties; Gao *et al.* created stable CDs with quenching response to Pt^2+^, Au^3+^, and Pd^2+^ ions.^102^ These dots benefitted from N-doping and possessed sensitive and low detection limits.

In an influential study, Ge *et al.* synthesized red CDs doped with sulfur and nitrogen which exhibit high sensitivity for detection of Fe^3+^ and whose fluorescence can be recovered.^20^ The addition of ascorbic acid to quenched CDs releases the –COOH and –SCN groups which Fe^3+^ binds to, thus allowing the CDs to regain most of their fluorescence. Another interesting application has been their use to detect mitoxantrone, an anticancer drug, in human serum. These red CDs exhibited a wide linear range of detection with low limit of detection, proving a use in ensuring proper drug administration.^103^ Additionally, these CDs were synthesized using citric acid for carbon and formamide for nitrogen, further showing the importance of precursor selection and surface modification.^104^ Other red CDs have been functionalized to detect gold ions as well as reducing them to Au^0^*via* redox with a surface thioether group on the CDs (Li *et al.*, 2022)^95^ at 29 nM, well below the. The detection *via* quenching began to occur at 29 nM, well below the reported 100 μM value at which a marked cell viability decrease occurs.^105^ The dual function of red CDs, in this case detecting and reducing ions, is often referred to as theranostics and will be discussed in detail later.

The detection capability of red CDs extends beyond ions and into pH sensing, which can play a variety of roles from spotting illnesses to imaging organelles. For example, Chen *et al.* synthesized red CDs which are responsive to pH change in the range of 4.0 to 8.0.^106^ These CDs shift color under ambient light from red to yellow as pH rises, and their fluorescence under UV also changes from purple to orange to yellow. Like many other reports, these dots possess nitrogen on their surface, showing the versatility of surface modification. Red CDs have further been fabricated whose responsiveness spans the range 1.0 to 9.0.^107^ These positively charged particles can enter lysosomes and decrease in fluorescence as pH increases. Nitrogen/sulfur co-doped CDs have been used to primarily detect extreme changes in pH, with higher emission corresponding to more acidic solutions.^108^ This study has also indicated that nitrogen and sulfur dopants play an important role in increasing QY and creating a reversible pH change detector. These studies, all relatively recent, indicate relatively novel and attractive potential for red CDs as pH sensors.

#### Theranostics

A novel field as well, theranostics represents a combination of diagnostic and therapeutic techniques, providing simultaneous imaging and treatment primarily for cancer. As the capabilities of red CDs for *in vivo* imaging, high permeability, and their biocompatibility have already been expanded upon, they act as an obvious candidate for theranostics. One common method is the concurrent use of imaging and photothermal therapy (PTT) in cancer cells. Due to the EPR of tumors and the small size of CDs, the dots naturally accumulate in cancer cells, making them advantageous for both imaging and treatment of diseases.^109^ CDs have been synthesized from a hydrophobic cyanine dye [2-((*E*)-2-((*E*)-2-chloro-3-((*E*)-2-(1-(2-hydroxyethyl)-3,3-dimethylindolin-2-ylidene)ethylidene)cyclohex-1-en-1-yl)vinyl)-1-(2-hydroxyethyl)-3,3-dimethyl-3*H*-indol-1-ium iodide, CyOH] and the addition of PEG800 to modify water solubility.^110^ These dots exhibit NIR fluorescence and, like the examples mentioned previously, have PTT higher than CDs on their own. However, surface modification has also been shown to increase the theranostic potential of red CDs. Getachew *et al.* were able to create a prodrug platform by doping red CDs with N, Cl, S, as well as Se, N, and CL, for on demand drug release and greatly improved optic qualities.^111^ This study depicts a major step forward in terms of chemotherapeutic red CDs, as it provided a greater level of control than many contemporary efforts.

Near-infrared CDs have been created which accumulated in tumors and provided PTT efficiency on par with current standards.^85^ These agents are advantageous due to their distinct NIR emission, providing stark contrast for imaging; in addition, these CDs were excreted renally almost entirely over the course of 24 hours. By preparation of polythiophene (PT2) and diphenyl diselenide in an alkaline solution, Lan *et al.* synthesized near-infrared CDs which proved function in two photon excitation imagery and in PTT.^21^ These dots surpass ordinary CDs in photothermal conversion efficiency due to their selenium and sulfur doping, which also provide wavelength independent NIR emission. As mentioned previously, CDs have been created with the curious ability to accumulate in mitochondria; these CDs also possess PTT potential as they generate ROS which trigger apoptosis (X.-Q. Wang *et al.*, 2023).^86^ These red-emitting CDs excite nearby oxygen when irradiated with a 635 nm laser and harm the mitochondria of the tumors in which they accumulate while also being potent imaging agents. Furthermore, scientists have functionalized near-infrared CDs into agents for photodynamic therapy and bioimaging, combining the imaging and treatment of tumors *in vivo*.^112^ These dots have advantageously high ROS generation and provide high-specificity imaging of tumors in mouse studies. Red-emitting CDs, relying heavily on formamide as a solvent, have been synthesized to exhibit both one-photon and two-photon fluorescence, further expanding their use in the field of imaging.^113^ These dots are also noteworthy for their ability to, upon photothermal activation, trigger cancerous cell death.

Surface modifications have also been used to functionalize CDs as nanoscale drug carrying devices. As mentioned previously, red CDs have been created from 4-aminophenol and potassium periodate which proved useful for delivery of doxorubicin.^88^ Doxorubicin was attached by addition in solution of PBS buffer, and the CDs absorbed it *via* π–π stacking interactions, indicating a simple procedure for drug loading. In addition, the CD–doxorubicin complex maintained its imaging use, displaying strong red emission in the nuclei of HeLa and breast cancer cells. The Cd–doxorubicin compound also proved to be much more effective than doxorubicin on its own, killing 79% *vs.* 50% of cancer cells. Additionally, CDs have been functionalized with cisplatin, another common chemotherapeutic, to minimize the effects of cisplatin on benign tissue.^62^ This CD–cisplatin complex accumulates in tumors, releasing the medicine and providing near-infrared emission for imaging. This molecule relies highly on surface modifications, as the CD–cisplatin complex must be coated in polyethylene glycol–chitosan–2,3-dimethyl maleic anhydride polymer (PEG–CS–DA) to achieve a negative surface charge; this prevents the adsorption of normal molecules like proteins during transit.^114^ Once the compounds reach tumor site, the slight acidity of the tumor flips the charge, releasing Pt(iv); high levels of glutathione in tumors then reduce this to Pt(ii), which is toxic and brings about tumor death. However, it also acts as a PTT agent, emitting enough heat when absorbing near-infrared light. This example is especially exciting due to its multifunctional nature and ability to reduce tumors in a variety of ways. One study by Li *et al.* created CDs which mimic large amino acids, enabling photoacoustic imaging and subsequent improved delivery of chemotherapeutics.^115^ This takes advantage of transporters like LAT-1, a technique which has been shown to increased accumulation of doxorubicin by at least threefold.^116^ Together with one of the aforementioned drug delivery methods, this could provide a novel and effective method for cancer inhibition.

#### Antibacterial uses

Due to the prevalence of bacterial infections worldwide, in both developed and developing nations, finding new ways to prevent and treat bacterial infections is of great importance.^117^ Gram-negative bacteria (like *Escherichia coli*) and drug-resistant bacteria are particularly difficult due to the severity of their infections and their ability to avoid antibiotics with the use of biofilms.^118,119^ As such, surface modified red-emitting CDs have been employed in a variety of antibacterial uses, helping to overcome issues like drug resistance or difficulties in detection of bacteria.^120^ These techniques mainly function in one of two different ways, either killing mature bacteria and biofilm, or inhibiting their growth and formation.^121^ Low cytotoxicity remains a primary goal for these antibacterial approaches, and the use of red CDs does not indicate significant cell death aside from the targeted bacteria.

Targeting mature bacteria and biofilm functions largely based on physical or oxidative damage to the bacteria. Interestingly enough, some of these approaches function similarly to many anti-cancer approaches, taking advantage of photothermal efficiency of CDs to target and kill drug-resistant bacteria. Mice with bacterial infections were treated photothermally with varying doses of red CDs and exhibited faster and fuller recoveries than their control groups, also showing no side effects in their organs.^120^ Even at low concentrations, CDs have been used to kill bacteria by destroying bacterial walls and inhibiting expression of genes.^122^ This broad-spectrum result is interesting not only because of its multifunctional mechanisms but also because it is self-degrading, preventing collateral damage *in vivo* and subsequent pollution.^123^ Nanoparticles for antibacterial use is not a novel field, as quantum dots and metal-based nanocomposites have demonstrated the ability to generate ROS in bacteria.^124,125^ However, CDs are unique in their ability to be synthesized from organic, and therefore safer, precursors, reducing harm to the environment and to non-bacterial cells.^126^

**Fig. 5:**
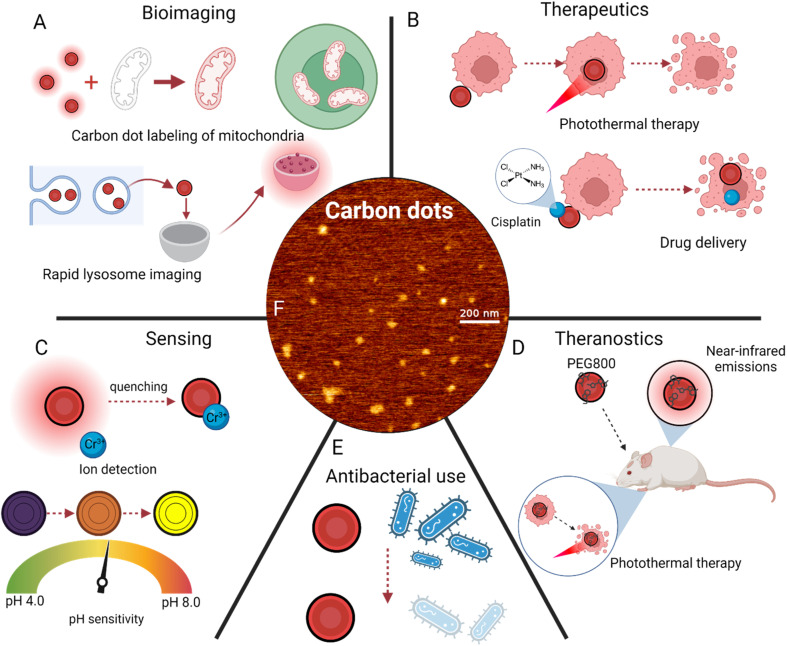
Applications of carbon dot surface modifications. (A) Bioimaging applications, including mitochondria labeling^74^ and lysosome imaging.^79^ (B) Disease treatment techniques, primarily photothermal therapy^21^ and drug delivery.^62^ (C) Sensing of ions^100^ and pH changes.^106^ (D) Theranostics, or simultaneous imaging and cancer treatment.^110^ (E) Antibacterial uses of red carbon dots. (F) Atomic force microscopy imaging of red carbon dots.^29^

While many approaches are currently focused on killing bacteria, there is also significant research behind red CDs for the prevention of bacterial growth. Studies on CDs have shown that the presence of surface groups improves their ability to inhibit bacteria growth.^127^ Going forward from this result, scientists have used N- and P-doped CDs for inhibiting the growth of *Staphylococcus aureus* and simultaneously detecting the potentially harmful Sudan Red I at low levels *via* fluorescence quenching.^128,129^ Additionally, nitrogen-modified red-emitting CDs have been used to inhibit progression of both cancerous cells and bacteria.^130^ Oxidative stress inducing DNA degradation was identified as the primary cause of bacterial inhibition, as the bacteria which could not develop properly was unable to spread.^131,132^ Interestingly, this study also revealed that monosaccharides as precursors had the best emission profiles among the 28 synthesized. Further pursuit of cheap and green methods of CD synthesis will doubtlessly benefit the field of antibacterial research (Fig. 5).

## Discussion

### Limitations in biological applications

Red CDs are evidently a powerful agent for applications ranging from imaging and sensing to therapeutics and antibacterial treatments. However, there are still significant issues that must be faced before their widespread adoption. Red CDs are produced with multiple different synthesis methods, most of which are bottom-up and thus less impactful on the environment.^133^ Additionally, these synthesis techniques often rely on cheaper materials and machinery, indicating a large degree of scalability. These greener methods, however, often lack as precise of control over size,^11^ indicating a need to hybridize with modern QD synthesis methods to balance lower cost with a higher degree of CD property control. One major issue faced by red CD synthesis is increasing the sensitivity of dots while maintaining a high degree of selectivity.^134^ Especially for sensing applications, it is hard to create CDs which have lower limits of detection without creating probes which are triggered by more ions than their desired target. Also, it is well reported that CDs should be kept below 10 nanometers in diameter to optimize their fluorescence and uptake properties.^135^ Because increasing particle size typically increases the wavelength emitted,^136^ it is a challenge to synthesize red CDs that remain below this size threshold. Additionally, surface functionalization often further increase size,^22^ making it of greater importance to better understand the role played by each part of the CD synthesis and modification process.

The processes of red CD synthesis and surface modification both rely on many different factors, with the main components being precursor choice, solvents used, pH, method of synthesis, and time and temperature used in synthesis.^38^ While versatility is something to celebrate in the stages of discovery, it is evident that synthesis and modification methods will need to become more streamlined and standardized before red CDs can be produced and used en masse. The precursor used has been shown to change many CD characteristics, most notably size and emission wavelength;^41,137^ using the right precursor can make the production of surface modified red-emitting CDs much easier. In addition, the solvent used has been demonstrated to impact quantum yield and emission wavelength,^24^ making it an important consideration during production. Solvent choice can also impact the outcome of surface modification methods, either by introducing new elements or preventing surface interactions.^22^ pH influences the color of CDs^138^ and their efficiency,^137^ both during production and in applications like pH sensing.^139^ Thus, the pH of solvent must be considered, and the pH range of the CD application must also be tested. As explained previously, there are many different methods which have successfully been used to synthesize red CDs.^140^ However, due to the effects that reaction time, pressure, and temperature^8,141^ play on the resultant CDs, it is important to choose a methodology that allows greater control; this is why bottom-up synthesis is increasingly common. As microwave synthesis, hydrolysis, and reflux have all created red CDs with modifications,^140^ these methods should be further studied so that specific, replicable protocol can be written. Without confirmation of the effects that each element in synthesis has on the final product, it is unlikely that red CDs will see implementation in a clinical setting.

In addition, a few issues present themselves due mainly to the novelty of CD research. While some studies have examined surface modification of CDs with bioagents, like antibodies,^56^ there is very limited research regarding specifically red CDs in this aspect. This likely stems from the fact that carbon dot structure is still not fully understood, and many professionals do not agree on the origin of their emissions.^142^ Current methods of characterization, like atomic force microscopy, reveal evidence of carbon dot formation but it is still unknown how atoms orient themselves at such a small level. As imaging advances, so will the understanding of the molecular interactions behind carbon dot structure. Finally, a major limitation is that, although carbon dots have proven anticancer behavior, they have yet to be tested clinically in humans. Obviously, this is a major roadblock which will be approached as the previously mentioned limitations are addressed, but it is important that further research regarding carbon dots and their surface modifications keeps clinical trials on the horizon.

### Prospects in translation

Red carbon dots, especially when surface modified, have a variety of appealing uses in medicine, ranging from bioimaging of specific organelles to therapeutics for diseases like cancer. However, there are significant limitations in current research, including poorly understood structure and a lack of defined synthesis procedures. As such, it is important to identify what should be focused on as carbon dot research progresses. One major medical challenge is crossing the blood–brain barrier, with implications ranging from Alzheimer's treatment to enhanced tumor targeting.^143^ The small size and easily modifiable surface nature of red carbon dots makes them particularly appealing. Also, since other synthesized nanoparticles have successfully crossed the blood–brain barrier,^144^ there is interest in doing the same with carbon dots. A more biocompatible, more sustainable nanocarrier, red CDs are seeing significant interest in the field of targeted medicine for brain conditions.

The field of chemotherapy is another target for red CDs as discussed previously. The CDs, through multiple studies, have shown increased uptake within tumor cells and high levels of subsequent tumorous cell death, all without reducing the viability of benign cells.^145^ In addition, surface modifications with molecules like dopamine have been beneficial to the compatibility of nanoscale chemotherapeutics.^146^ Compared to current chemotherapy regimens, which are plagued with off-target effects, this demonstrates a major step forward. Red CDs also solve the issue of imaging and therapeutics together, further reducing stress on patients and likely being a cost-effective method.^147^ However, before CDs see widespread adoption in this area, their synthesis methods need to be further refined in regard to their scalability and quality control.^148^

One major effort for translational chemotherapy is passive tumor targeting and subsequently triggered cell death, facilitated primarily by inorganic nanoparticles.^149^ For carbon dot clinical use, there are several precedents in modern chemotherapy that must be considered. Primarily, the small size of nanoparticles enables them to infiltrate tumors in higher quantities, preventing accumulation and further growth. Quantum dots and CDs, therefore, are an obvious candidate due to their ease of synthesis and small sizes.^150^ However, to prevent renal filtration and subsequent kidney damage, sizes are mostly kept above 10 nanometers.^151^ This stresses the importance of refined CD synthesis procedures, so that output size can be precisely monitored. However, since red CDs tend to have larger sizes corresponding with a larger wavelength, red CDs in specific have more potential use. Another important factor in clinical translation is that most nanoparticles in use currently are inorganic, with gold being a forerunner.^152,153^ This can have unintended cytotoxic effects, putting further stress on the bodies of cancer patients.^154^ Organic-based nanoparticles, therefore, should be further developed so that their non-toxic nature can be fully used.

The second main method of chemotherapy is targeted treatment by means of photothermal and photodynamic therapy. Due to the unique characteristics of high quantum yield and excellent photothermal conversion,^116^ quantum dots have been used *in vivo* and *in vitro* to reduce tumor size. Their luminescence also allows easy tracking and subsequent imaging of the successes or failures of treatment.^155^ To maintain sufficiently large size, as mentioned above, carbon dot aggregates have been recently created, with an added initiative of improved quantum yield.^156^ This approach synergizes well with longer wavelength CDs, as the aggregation further boosts their photothermal conversion efficiency, and longer wavelengths are shown to penetrate tissue more.^157^ This property of long-wavelength light has led to significant efforts to tune CD emissions towards the near-infrared region.^158^ By attaching surface molecules and adjusting precursors, more effective photothermal agents can easily be developed, indicating the large potential for clinical use of red, surface modified CDs.^159^ The main drawback noted in current photothermal/photodynamic chemotherapy approaches is phototoxicity, which is a negative reaction of tissue to the light that is required to activate PTT agents.^160,161^ CDs have been functionalized with Chlorin-e6 through amide reaction to reduce phototoxicity when activated with a laser.^162^ This novel method also yielded simultaneous photothermal therapy and photodynamic therapy under one-laser activation, presenting a safer and simpler process for targeted chemotherapy. As efforts continue to reduce phototoxicity, red CDs could see clinical use in conjunction with other medications for a holistic approach to cancer treatment.

Finally, as mentioned previously, efforts have been made to use CDs as drug delivery devices to treat tumors in a localized manner.^163^ Because red CDs are the largest of their family,^164^ it makes sense to use them to encapsulate drug molecules or bond with them on their surface. Dopamine has been successfully electrostatically conjugated to the surface of red CDs, increasing uptake *in vivo* and reducing cancer cell viability by as much as 50%.^29^ In addition, doxorubicin has been attached to CDs for delivery to human breast cancer cells.^59^ Obviously, one major drawback to drug delivery CDs is that their novelty prevents them from experiencing any clinical trials. As there are many different approaches yielding varying degrees of chemotherapeutic success, further research will be required before a consensus is reached on the optimal path to carbon-based drug delivery.^165^ This method also suffers from additional required focus on cytotoxicity and side effects, as accumulation of both CDs and the associated drug must be monitored and controlled.^166^ An interesting study managed to reduce cytotoxicity by embedding CDs in a PEG matrix and subsequently using them for chemotherapy.^167^ This method, however, works by reducing the ROS generation from CDs, eliminating its use for passive targeting chemotherapy or photothermal therapy. Still, it is important to note that efforts are being made to improve safety along with efficiency for translational red CD use.

## Conclusion

The study of carbon dots has already progressed greatly, from an accidental discovery to an emerging prospect for bioimaging and drug delivery. Red carbon dots provide distinct imaging advantages due to high contrast and ease of surface modification. A variety of methods exist to modify carbon dots, each with its own advantages and applications. Further focus on red carbon dot modification to increase their uptake in specific cell types/organs, as well as development of a well-understood synthesis process, will ensure that surface-modified red carbon dots remain at the forefront of biomedical research. In focusing on clinical translation of carbon dot treatment regimens, it is important to thoroughly understand the drawbacks and positive traits of carbon dots. As they remain a novel nanomaterial, this will undoubtedly come with continued research of reduced cytotoxicity, enhanced targeting, and better control of size during synthesis.

## Conflicts of interest

No conflicts of interest were declared by the authors.

## Supplementary Material
